# Body composition and serum levels of adiponectin, vascular endothelial growth factor, and interleukin-6 in patients with rheumatoid arthritis

**DOI:** 10.3325/cmj.2012.53.350

**Published:** 2012-08

**Authors:** Sergey P. Oranskiy, Ludmila N. Yeliseyeva, Anna V. Tsanaeva, Nadezhda V. Zaytseva

**Affiliations:** Kuban State Medical University, Krasnodar, Russian Federation

## Abstract

**Aim:**

To investigate differences in body composition and body mass index (BMI) in patients with rheumatoid arthritis (RA) and their correlations with serum production of adiponectin, interleukin-6 (IL-6), and vascular endothelial growth factor (VEGF).

**Methods:**

The study included 83 patients (age 53 ± 5 years) with RA treated with methotrexate. We determined their BMI, fat mass, and fat-free mass using bioimpedance analysis, and serum concentrations of adiponectin, VEGF, and IL-6 using immunoassay analysis.

**Results:**

Normal BMI was found in 39 (47%), overweight and obesity in 26 (31%), and underweight in 18 (22%) patients. Concentration of adiponectin was lower in overweight/obese patients than in patients with normal BMI (2.1 [0.8-3.9] μg/mL vs 8.9 (7.2-11.3) μg/mL). In underweight patients, it was moderately increased (12.7 [9.3-14.8] μg/mL) and the correlation between the concentrations of adiponectin and IL-6 was positive (r = 0.4; *P* = 0.01). Concentrations of VEGF and IL-6 were increased in all groups with RA. The overweight/obese group showed a negative correlation between the concentrations of adiponectin and VEGF (r = - 0.34; *P* = 0.04), a positive correlation between VEGF concentration and fat mass (r = 0.39; *P* = 0.02), and a negative correlation between adiponectin concentration and fat mass (r = - 0.23; *P* = 0.02).

**Conclusion:**

Inflammatory and angiogenesis activation was found in RA patients with all types of body composition, but only in those with obesity and overweight there was a direct antagonism between adiponectin and VEGF. Further research is needed to identify possible regimens of metabolic correction in different variations of body composition.

Rheumatoid arthritis (RА) is one of the most frequent autoimmune diseases, with up to 1.3% prevalence in the world, which is also associated with a high risk of disability (1). The leading cause of death in RA patients are cardiovascular diseases (2,3), which can be related to changes of nutritional and metabolic status and correlate with body mass index (BMI). However, little is known about the impact of different metabolic factors on body composition in RA patients (4,5).

According to the World Health Organization (WHO) criteria, there are three types of metabolic phenotypes as determined by BMI – normal BMI, underweight, and obesity (6). WHO criteria do not embrace the term “cachexia,” but this is one of the most discussed problems in patients with RA. One of the latest definitions was: “Cachexia is a complex metabolic syndrome associated with underlying illness and characterized by loss of muscle with or without loss of fat mass” (7), but there is no generally accepted definition of cachexia in RA patients. Cachexia in RA is divided into two types – the “classic” low-BMI type (with both low muscle mass and low fat mass) and the more frequent type termed “rheumatoid cachexia,” associated with normal or increased BMI (characterized by low muscle mass but increased fat mass) (8). Many RA patients with or without cachexia have protein energy insufficiency and reduction of muscular and/or fat mass, which is connected with a hypercatabolism state and proinflammatory activation (9). The problem of metabolic syndrome and obesity in patients with RА has recently attracted more research attention (10) and it seems that the frequency of these conditions is quite high (11).

Metabolic changes can be associated with the effects of adipokines. One of the key adipokines is adiponectin, a cytokine-like protein hormone and one of the main regulators of carbohydrate and fat exchange, produced exclusively in the fat tissue. Adiponectin has structural amino acid sequences homologous with collagens, some complement factors, and tumor necrosis factor-α (12). Decreasing adiponectin production leads to insulin resistance, because of its ability to inhibit gluconeogenesis and to stimulate oxidation of free fat acids in skeletal muscles and the liver (13). So, the adiponectin level decreases in patients with obesity, metabolic syndrome, type II diabetes mellitus, and cardiovascular diseases (14). In autoimmune diseases, adiponectin acts also as a cytokine regulator with functional dualism and the ability to influence proinflammatory and anti-inflammatory mediators (cytokines, growth factors, etc.) (15,16).

Increased production of serum adiponectin and correlation of adiponectin with proinflammatory cytokines was found in RА patients with normal body weight (17,18), but only one study investigated the production of serum adiponectin in RA patients with obesity (19). Also, most studies identified increased serum concentrations of IL-6 and VEGF in RA patients with normal body weight (20-22), without studying the patients with reduced or increased BMI. Choi et al (18) found a direct effect of adiponectin on the stimulation of VEGF in the synovial fluid of RA patients. Serum concentrations of adiponectin, VEGF, and IL-6 have not yet been investigated in RA patients with different BMI and body composition variations. The aim of this study was to investigate differences in body composition and BMI in patients with RA and their correlations with serum production of adiponectin, IL-6, and VEGF.

## Materials and methods

The study included 83 patients admitted to the Rheumatology Department of Kuban State Medical University and Krasnodar Regional Hospital (15 men, 68 women) with seropositive RА. They were divided into 3 groups: patients with normal BMI (n = 39), patients with obesity and overweight (n = 26), and patients with underweight (n = 18). Patients with obesity and overweight were fused in one group due to their similar effect on serum adiponectin and proinflammatory cytokine status in general population (23). The control group (20 persons – 4 men and 16 women) was formed from healthy blood donors and randomized with RA groups using stratified randomization by age and sex. RA groups and the control group did not differ according to demographic characteristics. RA groups did not differ according to clinical characteristics and medications (Table 1). We excluded the patients with concomitant infectious, oncological diseases, purulent conditions of any localization, and those with renal, hepatic, or cardiac insufficiency. To eliminate the influence of nutritional factors on BMI and body composition we used the Mini Nutritional Assessment (24). There were no differences between the groups of RA patients according to all indicators of this questionnaire. The majority of RA patients received methotrexate as a disease-modifying drug, without differences in dosages and duration of therapy between RA groups. Also, to avoid the influence of symptomatic therapy on body composition, we retrospectively quantified the cumulative dosage of nonsteroidal anti-inflammatory drugs (NSAID) and corticosteroids. Cumulative dosage was calculated as: daily dosage × number of days of dosing. We did not register any differences in duration and cumulative dosage between RA groups. Body composition indicators – BMI, fat mass, and fat-free mass – were determined using bioimpedance analysis (TANITA BC-418 analyzer, Tanita Corporation, Tokyo, Japan). The study was approved by the Ethics Committee of the Human Studies of Kuban State Medical University (Krasnodar, Russia) and was conducted according to principles of the Helsinki Declaration of the World Medical Association (2008 revision). Informed consent was received from all patients and the control group.

**Table 1:** Basic demographic and clinical characteristics of the study cohort*

Parameter	Controls (n = 20)	Patients with normal BMI (n = 39)	Patients with obesity and overweight (n = 26)	Underweight patients (n = 18)
Percent of women	80.0	82.0	80.0	86.0
Age in years (median and range)	52.0 (50.0-60.0)	53.0 (51.0-62.0)	52.0 (50.0-57.0)	55.0 (51.0-59.0)
Current smoking, n (%)	3.0 (15.0)	6.0 (15.4)	4.0 (15.4)	3.0 (16.7)
Duration of RA in years (median and range)	-	6.5 (4.5-9.0)	6.0 (5.0-9.0)	6.7 (5.4-10)
Rheumatoid factor positive, n (%)	-	83.0	80.0	79.0
ACCP positive, n (%)	-	34.0 (87.0)	23.0 (89.0)	15.0 (83.0)
CRP, mg/L (median and range)	-	20.5 (14.9-20.2)	20.0 (14.0-28.0)	22.0 (13.0-29.0)
ESR in mm/h (median and range)	8.0 (2.0-10.0)	33.5 (26.0-37.5)	20.0 (14.0-28.0)	29.0 (15.0-38.0)
DAS28 (median and range)	-	7.3 (6.3-7.8)	7.2 (6.9-7.7)	7.4 (6.2-8.1)
Current methotrexate, n (%)	-	30.0 (77.0)	21.0 (81.0)	14.0 (78.0)
Current prednisone, n (%)	-	24.0 (62.0)	18.0 (69.0)	13.0 (72.0)
Current NSAIDs, n (%)	-	15.0 (38.0)	10.0 (42.0)	8.0 (44.0)

*Abbreviations: BMI – body mass index; RA – rheumatoid arthritis; ACCP – antibodies to cyclic citrullinated peptide; CRP – C-reactive protein; ESR – erythrocyte sedimentation rate; DAS28 – disease activity score-28; NSAID – non-steroidal anti-inflammatory drug.

Patients were admitted to our research unit between September 12, 2009 and September 12, 2011, and a detailed history and physical examination was taken. We assessed the disease activity with the Disease Activity Score (DAS28), including tender and swollen joint count and general health status (25). After overnight fasting, hemogram and leukocyte count, erythrocyte sedimentation rate (ESR), elemental serum biochemistry (electrolytes, urea, creatinine, albumin, standard liver function tests, transaminases, bilirubin, albumin), rheumatoid factor (RF), and C-reactive protein (CRP) were determined. Blood samples were collected in sterile Vacutainer tubes, centrifuged at 3500 rpm for 15 minutes at 4°C, and stored at -80°C in pyrogen-free polyethylene tubes. Body mass index (BMI) was calculated according to the formula: [body weight/height^2^] (kg/m^2^). Due to the lack of generally accepted criteria for rheumatoid cachexia, we divided the patients with RA according to the WHO criteria (6). In accordance with the WHO standards, patients with BMI values lower than 18.5 kg/m^2^ were classified as underweight, with values between 18.5 and 24.9 as normal, with values between 25-29.9 kg/m^2^ as overweight, and with values greater than 30 as obese.

We measured the serum concentration of adiponectin, VEGF, and IL-6 using the BioVendor (Modrice, Czech Republic), Invitrogen (Camarillo, CA, USA), and Protein Contour Ltd (Saint-Petersburg, Russian Federation) kits and using immune-enzyme analysis (analyzer Statfax 2100, Awareness Technology Inc., Palm City, FL, USA). All data were processed using Statistica 6.0. (Statsoft, Tulsa, OK, USA). Considering the asymmetrical shape of distribution (Kolmogorov-Smirnov test), data were presented as medians and ranges. Differences between unpaired samples were measured with the nonparametric Mann-Whitney test. We also performed a correlation analysis between all parameters studied in patients with RA. Correlations were determined with the Spearman rank order correlation test.

## Results

Patients in all groups had medium disease activity according to general RA markers – DAS28, ESR, CRP, and most of them were treated with methotrexate as disease-modifying drug. There were no differences in duration, dosage of corticosteroids, or NSAIDs treatment (Table 1).

Thirty nine (47%) patients had normal BMI, 26 (31%) were obese and overweight, and 18 (22%) were underweight (Table 1). There were no differences in fat mass and fat-free mass between patients with normal BMI and the control group. Only overweight and obese patients had high fat mass and low fat-free mass. This means that this group presented with rheumatoid cachexia. Underweight group had lower fat mass and lower fat-free mass (classic low BMI type of rheumatoid cachexia) than the control group and the group with normal BMI (Table 2).

**Table 2:** Body composition of the study cohort patients*

Indicator (median and range)	Control group (n = 20)	Patients with overweight and obesity (n = 26)	*P*	Underweight patients (n = 18)	*P*	Patients with normal BMI (n = 39)	*P*
Body mass index (BMI), kg/m^2^	23 (21.2-24.1)	31.8 (29.5-40.1)	0.04^†^; 0.04^‡^	17.9 (16.5-19.2)	0.03^†^; 0.04^‡^	25.2 (24.1-27.3)	0.07^‡^
Fat mass, kg	19.3 (17-22)	29.1 (26.5-31.2)	0.04^†^; 0.03^‡^	17.5 (14.6-19)	0.04^†^; 0.04^‡^	21.2 (19-25.4)	0.06^‡^
Fat mass, %	25.3 (20.2-31.9)	38.9 (37.0-40.0)	0.04^†^; 0.02^‡^	18.9 (18.4-23.5)	0.02^†^; 0.03^‡^	28.7 (22.9-30.7)	0.07^‡^
Fat free mass, kg	53.2 (51.4-68.6)	51.0 (49.6-59.5)	0.04^†^;0.04^‡^	45.1 (37.6-46.4)	0.03^†^; 0.03^‡^	55.1 (52.4-57.9)	0.06^‡^
Fat free mass index, kg/m^2^	18.2 (16.9-19.4)	17.3 (16.2-19.1)	0.04^†^; 0.04^‡^	15.6 (14.5-16.9)	0.04^†^; 0.04^‡^	19.3 (18.3-20.6)	0.07^‡^

*Significance of differences calculated with the Mann-Whitney test.

†*P* value of differences with the group of patients with normal BMI.

‡*P* value of differences with the control group.

The concentration of adiponectin was higher in the group with normal BMI than in overweight and obese patients (8.9 [7.2-11.3] vs 2.1 [0.8-3.9] μg/mL) and significantly lower than in underweight patients (12.7 [9.3-14.8] μg/mL). The concentration of IL-6 was higher in all groups with RА than in controls; however, in the group with obesity it was higher than in the group with normal BMI, and in the group with normal BMI it was higher than in underweight patients (Table 3). Underweight patients had a positive correlation between adiponectin and IL-6 serum concentrations (r = 0.4; *P* = 0.01).

**Table 3:** Serum concentration of adiponectin, interleukin-6, and vascular endothelial growth factor in study patients*

Parameter(median and range)	Control group (n = 20)	Patients with overweight and obesity (n = 26)	*P*	Underweight patients (n = 18)	*P*	Patients with normal body mass index (n = 39)	*P*
Adiponectin, μg/mL	6.8 (3-7.1)	2.1 (0.8-3.9)	0.01^†^; 0.03^‡^	12.7 (9.3-14.8)	0.03^†^; 0.01^‡^	8.9 (7.2-11.3)	0.06^‡^
Interleukin-6, pg/mL	5.0 (2.3-9.9)	57.3 (50.6-73.3)	†0.03^†^; 0001^‡^	25.2 (18.8-32.9)	0.03^†^; 0.001^‡^	42.4 (29.3-53.9)	0.02^‡^
Vascular endothelial growth factor, pg/mL	20.0 (12.3-39.4)	175.0 (171.8-203.9)	†0.02^†^; 0.002^‡^	119.0 (108.1-169.2)	0.02^‡^	111 (73.1-158)	0.02^‡^

*Significance of differences calculated with the Mann-Whitney test.

†*P* value of differences with the group of patients with normal BMI.

‡*P* value of differences with the control group.

The concentration of VEGF was higher in all RA groups than in the control group. However, the group with obesity and overweight had higher VEGF values than the normal BMI and control group (Table 3). The group with obesity and overweight had a negative correlation between adiponectin and VEGF concentrations (r = -0.34; *P* = 0.04), a positive correlation between VEGF concentration and fat mass (r = 0.39; *P* = 0.02), and a negative correlation between adiponectin concentration and fat mass (r = - 0.23; *P* = 0.02).

## Discussion

In our study, patients with overweight and obesity had rheumatoid cachexia, with reduced fat-free mass and increased fat mass. The underweight group had classic low BMI type of rheumatoid cachexia. This is in accordance with the findings of Elkan et al (9), except that in our study patients with normal BMI had no rheumatoid cachexia.

Adiponectin production in adults has been found to inversely correlate with body fat percentage (26), but the data in patients with RA are limited. Adiponectin is known to play an important role in the pathogenesis of RA, although its action as an anti-inflammatory or pro-inflammatory mediator is controversial (14-16). In general population, low adiponectin levels have been associated with obesity, type 2 diabetes mellitus, atherosclerosis, and metabolic syndrome, and its role in these conditions is clearly anti-inflammatory (27-29). On the other hand, some authors have reported high adiponectin levels correlating with disease activity in general population of RA patients (30). Only one study (19) demonstrated hypoadiponectinemia in patients with RA and obesity, which is similar to our data. In our study, serum concentration of adiponectin increased in RA patients with normal BMI and underweight patients and decreased in obesity/overweight patients.

Another important aspect of adiponectin production is the potential impact of different local (joint) and systemic (serum) effects. At the joint level, adiponectin contributed to synovitis and joint destruction in patients with RA (without specifying body composition type) by stimulating VEGF and matrix metalloproteinases expression in fibroblast-like synoviocytes (18). We found a negative correlation between adiponectin and VEGF serum concentration in obese/overweight patients and a positive correlation between adiponectin and IL-6 serum concentration in underweight patients. Until recently, data about the link between VEGF, other angiogenic factors, and cardiovascular risk were contradictory. A recent study has demonstrated an association between VEGF gene polymorphisms and RA (31). Another study has found no significant association between the VEGFA rs2010963 and s1570360 polymorphisms and clinically evident cardiovascular disease (32). In yet another study, levels of VEGF have been negatively associated with 10-year cardiovascular disease and stroke risk (33). There are no unanimous data on the concentration of IL-6 in different types of body composition in the general population of RA patients. In our study, serum concentration of VEGF and IL-6 was increased in all types of body composition. We also found a lower level of IL-6 in underweight patients than in normal BMI and overweight patients. IL-6 level in RA patients may be related to genetic polymorphism as well. In patients with RA associated with obesity or smoking, IL-6 levels were significantly increased in C-allele carriers, but no significant interactions were recorded between adiposity and IL6-174G/C genotypes (34).

We registered all types of body composition and BMI variations (normal BMI, overweight/obesity, and underweight) in RA patients. Patients with overweight/obesity and underweight had rheumatoid cachexia. All BMI variations were associated with IL-6 and VEGF activation but only in the group with overweight and obesity we found correlations between fat mass and serum concentrations of adiponectin and VEGF, which may point to a more active stimulation of angiogenesis and vasculature and inhibition of a key adipocytokine. Further research is required to identify possible regimens of metabolic correction in different variations of body composition.
